# Dosimetric feasibility of stereotactic arrhythmia radioablation for ventricular tachycardia in patients with a subcutaneous implantable cardioverter defibrillator

**DOI:** 10.1016/j.phro.2025.100827

**Published:** 2025-08-21

**Authors:** Lena Kaestner, Jingyang Xie, Hannah Fanslau, Kerstin Siebenlist, Miriam Eckl, Hans Oppitz, Jens Fleckenstein, Daniel Buergy, Mustafa Kuru, Jürgen Kuschyk, Daniel Dürschmied, Mathieu Kruska, David Krug, Frank A. Giordano, Achim Schweikard, Oliver Blanck, Boris Rudic, Judit Boda-Heggemann

**Affiliations:** a Department of Radiation Oncology, University Medical Center Mannheim, Medical Faculty Mannheim, University of Heidelberg/ DKFZ Hector Cancer Institute at the University Medical Center Mannheim, Theodor-Kutzer-Ufer 1-3, 68167 Mannheim, Germany; b Institute for Robotics and Cognitive Systems, University of Luebeck, Ratzeburger Allee 160, 23562 Lübeck Germany; c Department of Radiology, University Medical Center Mannheim, University of Heidelberg, Theodor-Kutzer-Ufer 1-3, 68167 Mannheim, Germany; d I. Department of Medicine: Cardiology, Angiology, Hemostaseology and Intensive Care, University Medical Center Mannheim, Medical Faculty Mannheim, University of Heidelberg/ DZHK (German Centre for Cardiovascular Research) Partner Site Mannheim, Theodor-Kutzer-Ufer 1-3, 68167 Mannheim, Germany; e Department of Radiation Oncology, University Hospital Hamburg-Eppendorf, Martinistrasse 52, 20246 Hamburg, Germany; f Department of Radiation Oncology, University Medical Center Schleswig-Holstein, Arnold-Heller-Straße 3, 24105 Kiel, Germany

**Keywords:** STAR (stereotactic arrhythmia radioablation), Subcutaneous implantable cardioverter defibrillator, Planning study

## Abstract

**Background and purpose:**

Subcutaneous implantable cardioverter defibrillators (S-ICDs) are increasingly used to prevent sudden cardiac death. Due to their placement near the left lateral and apical myocardium, S-ICDs can present technical challenges for stereotactic arrhythmia radioablation (STAR) of ventricular tachycardia. This study aimed to evaluate the feasibility of STAR in patients with S-ICDs.

**Materials and methods:**

Previously acquired cardiac computed tomography data from ten S-ICD patients were retrospectively analyzed. Organs at risk, S-ICD components, and hypothetical planning target volumes (PTV) for segments 8 (septal), 11 (lateral), and 17 (apical) were contoured. For each patient and segment, two treatment plans were calculated with a prescribed PTV dose of 25 Gy: one plan sparing the S-ICD from the primary radiotherapy beam (‘spared’) and one without this constraint (‘non-spared’).

**Results:**

The S-ICD was successfully spared from the primary beam in 27 out of 30 plans, with *D*_0.035cm_^3^ < 1 Gy. In three spared plans (PTV segment 11) with a distance between PTV and S-ICD <4 cm, *D*_0.035cm_^3^ exceeded 1 Gy. Spared plans for PTV segment 11 showed significant higher dose/volume metrics and reduced plan quality compared to non-spared plans (p < 0.05), although 9/10 plans remained clinically acceptable. For PTV segment 8 and segment 17, no clinical or statistical differences were observed.

**Conclusions:**

The presence of an S-ICD is not a contraindication for STAR, although plan quality may decrease with PTVs in lateral segments. Rare cases with a distance between PTV and S-ICD <4 cm may complicate radiation treatment planning.

## Highlights

• Creation of three cardiac targets in ten patients with subcutaneous defibrillators.
• Subcutaneous defibrillators were spared in 27 out of 30 cardiac radioablation plans.
• A subcutaneous defibrillator is no contraindication for cardiac radioablation.
• Mainly for lateral targets, subcutaneous defibrillators can reduce plan quality.
• Anatomical variations (target and defibrillator <4 cm) may complicate planning.

## Graphical abstract

**Figure:**
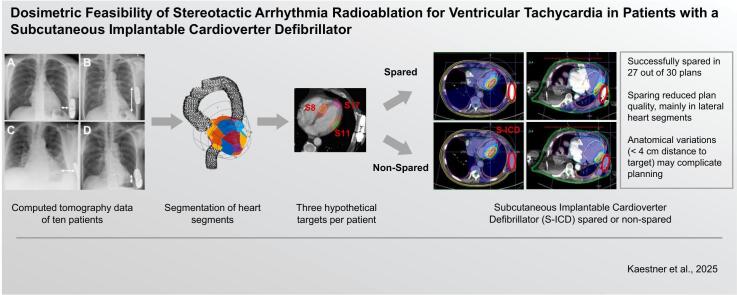

## 1 Introduction

**Fig. 1:**
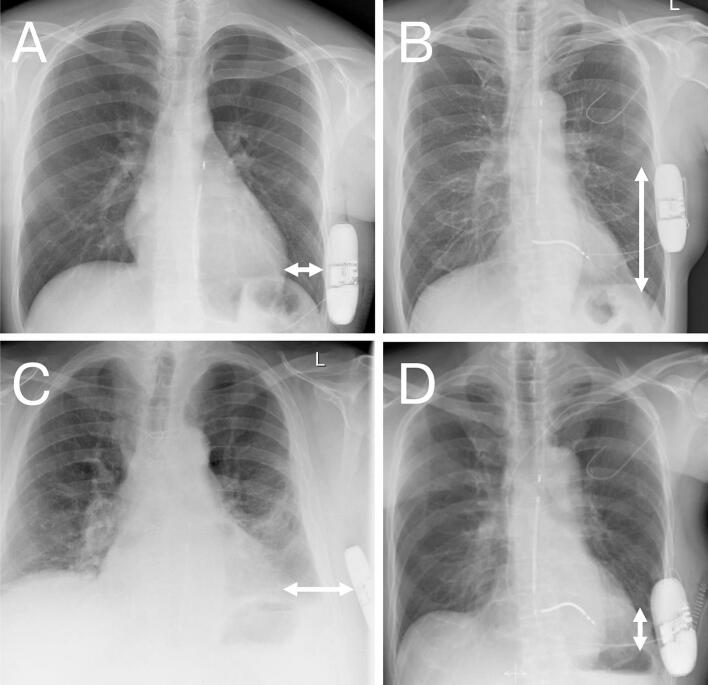
S-ICDs localized in the left axillary region (sub-serratus, intermuscular, and subcutaneous placement). Exemplary X-rays of S-ICD patients treated in our clinic. Depending on patient anatomy, varying distances between S-ICD and left ventricle occur: A) Normal-weight patient with a small distance between S-ICD and the heart in the left–right direction. B) Patient with a cranially implanted can and a resulting large craniocaudal distance between the S-ICD and the heart. C) Obese patient with a large distance between the S-ICD and the heart in the left–right direction. D) Patient with a caudal can placement and a resulting small craniocaudal distance between the S-ICD and the heart.

The subcutaneous implantable cardioverter defibrillator (S-ICD) is widely used to prevent sudden cardiac death of patients with ventricular tachycardia. Several prospective trials have demonstrated its efficacy and established it in current guidelines [1,2]. Its extravascular design avoids direct contact with the heart and vasculature, reducing complications such as lead fractures, infections, and venous occlusion. It is particularly suitable for patients with difficult venous access or increased infection risk. Unlike transvenous ICDs, the S-ICD generator is implanted between the latissimus dorsi and serratus anterior muscle in the left midaxillary line, typically between ribs five and six. The defibrillation coil runs parallel to the left of the sternum and ends near the sternal notch. Implantation positions vary considerably in all anatomical planes, resulting in varying distances to the heart (Fig. 1).

As S-ICDs are used more frequently, studies report appropriate shocks in up to 16 % of patients within four years [3,4]. Recurrent ventricular tachycardia and device shocks reduce quality of life and are linked to higher mortality [5]. Radiofrequency catheter ablation is the gold standard for symptomatic ventricular tachycardia, reducing both symptoms and mortality [6,7]. However, long-term recurrence rates can exceed 50 %, reflecting the limited ability of radiofrequency energy to modify transmural arrhythmogenic substrates [8].

Stereotactic arrhythmia radioablation (STAR) has emerged as a non-invasive treatment option for therapy-refractory ventricular tachycardia [[9], [10], [11], [12]]. By enabling transmural effects deep in the myocardium, STAR addresses the limitations of catheter ablation in creating durable lesions at critical substrate sites [10]. Studies suggest that a single 25 Gy fraction is effective in inducing protein expression changes, conduction slowing, and likely fibrosis with conduction block – suppressing reentrant ventricular tachycardia [13,14].

Radiotherapy may directly affect cardiac implantable electronic device components and function [15]. Malfunction is suspected at doses ≥2 Gy [16], although threshold recommendations remain debated [17,18]. The impact of STAR on ICD function is still under investigation, but initial data show no clinically relevant effects on devices or leads [15]. In contrast, reduced battery longevity was observed in an in-vitro lung model after 18 Gy in three fractions, with a maximum S-ICD dose of 3.5 Gy [19]. Consequently, STAR trial protocols often limit cardiac implantable electronic device doses to 0.5 Gy (and, as an acceptable deviation, 1 Gy) [20,21], although most are based on conventional ICDs.

By design, S-ICDs are implanted closer to the heart, particularly the anterolateral left ventricular wall. As STAR is typically planned using coplanar volumetric arc therapy, the device often lies within the same transverse plane as the target, increasing potential exposure.

STAR is currently being investigated in clinical trials and occasionally used as palliative therapy [22,23]. With a growing number of STAR procedures – including in S-ICD carriers – the need for guidance on safe treatment planning becomes increasingly relevant. To our knowledge, STAR treatments have already been performed in at least two S-ICD patients within the STOPSTORM consortium [24] and one was treated in our clinic. However, no dedicated treatment planning study exists.

In this retrospective treatment planning study, cardiac computed tomography (CT) scans from S-ICD patients were used to simulate radioablation plans for representative LV segments. Plans were designed to either spare the device from the primary beam (‘spared’ protocol) or to optimize target coverage without considering device location (‘non-spared’ protocol). Resulting plans were analyzed regarding dose constraints, device-target anatomy, and plan quality. The objective of this study was to assess the feasibility and applicability of STAR in patients with an S-ICD.

## 2 Materials and methods

### 2.1 Cardiac CT scan for planning simulation

Previously acquired clinical and cardiac contrast-enhanced CT data (slice thickness ≤3 mm) of ten S-ICD patients (seven male, three female, median age of 76 years [range, 52–86 years]) were retrospectively utilized for STAR treatment planning with simulated ventricular tachycardia targets based on the American Heart Association 17-segment model after local ethics board approval (2020-856R). As all CT scans of S-ICD patients had originally been acquired for other indications, patient selection was based on visibility of heart and S-ICD on the CT scan as well as a CT slice thickness below 3 mm.

### 2.2 Segmentation

**Fig. 2:**
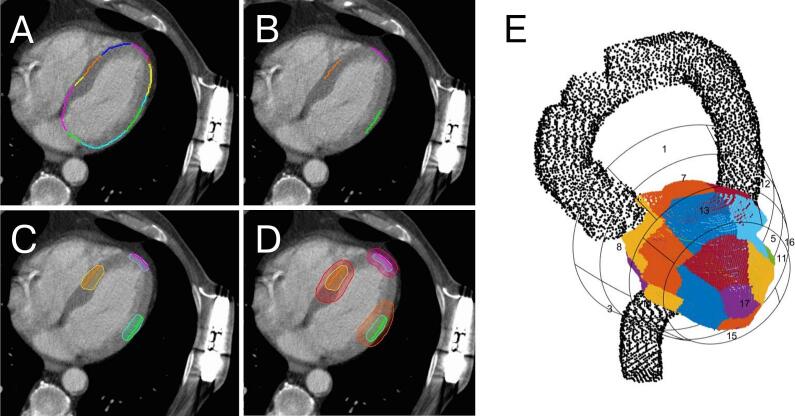
Creation of hypothetical targets in three representative heart segments. A) Semi-automated transfer of all 17 segments (American Heart Association) of the left ventricle with CARDIO-RT to the cardiac CT (transversal slice shown, segments in assorted colors; S-ICD left axilla). B) The representative segments 8 (septum, orange), 11 (left lateral, green), and 17 (apical, magenta). C) Transmural expansion to CTVs of the three representative segments. D) Final PTV (CTV + 5 mm). E) Volume rendering of the left ventricle and the semi-automatically transferred 17 segments (CARDIO-RT). Black: aorta. (For interpretation of the references to colour in this figure legend, the reader is referred to the web version of this article.)

Three hypothetical representative STAR targets for septal, lateral, and apical left-ventricular regions were created for each patient: Individual CT scans were semi-automatically segmented with the software CARDIO-RT (University of Lübeck, Germany) [25,26], according to the standardized American Heart Association 17-segment model. Segments (S) 8, 11, and 17 were identified as segments representing the septal, lateral, and apical left-ventricular regions. Based on the contoured/transmurally expanded segments as clinical target volumes (CTV), three planning target volumes (PTV) for S8, S11, and S17 were created for each patient in a radiotherapy imaging system (Velocity V.4.1, Varian, Palo Alto, USA; Fig. 2) with a 5 mm CTV-PTV margin (RAVENTA study [RAdioablation for VENtricular Tachycardia, NCT03867747] protocol) [20,22,27]. Additionally, S-ICD components (device and shock coil), cardiac (sub)structures (whole heart, left anterior descending artery [LAD]), and organs at risk (aorta, thoracic wall, heart-PTV, LAD, lungs, spinal canal, skin, esophagus/stomach) were delineated according to best practice guidelines [28].

### 2.3 Treatment planning

**Fig. 3:**
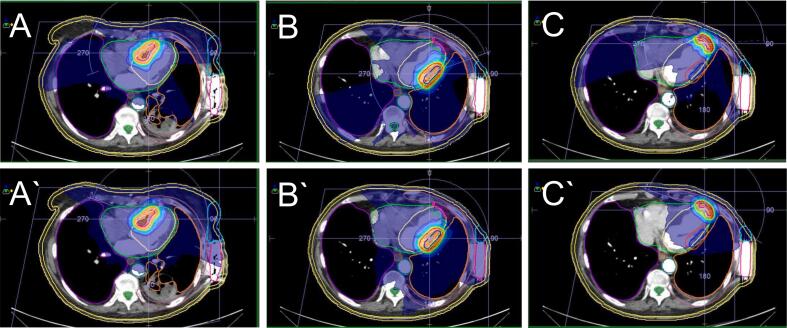
Exemplary treatment plans of one case. A-C) Radiotherapy plans (‘spared’) with 25 Gy in one fraction for the PTV of segment 8, 11, and 17 with sparing of the S-ICD from the primary beam (note the arc configuration). A‘-C‘) Radiotherapy plans for the PTV of segment 8, 11, and 17 without sparing of the S-ICD from the primary beam (‘non-spared’). For this patient, sparing of the S-ICD did not result in plan quality compromises in any of the cases.

For each patient, six volumetric arc therapy plans (6 MV, flattening-filter-free, 2 mm calculation grid size, 5 mm leaf thickness [Elekta Agility Collimator], Monaco V6.1, Elekta AB, Sweden) with three coplanar arcs and a PTV dose of 25 Gy according to the RAVENTA study protocol (dose coverage PTV recommended >90 %, optimal 95 %) and best practice recommendations [29] were created: In three treatment plans (one for each S8, S11, and S17) the S-ICD (recommended *D_max_* < 1 Gy, optimal <0.5 Gy) was spared from the primary treatment beam (= ‘spared’ plan). In the other three treatment plans (one for each S8, S11, and S17), the S-ICD was not spared from the primary treatment beam (= ‘non-spared’ plan). That way, a total of 60 treatment plans for ten patients with hypothetical target volumes in three segments (S8, S11, S17) were created for this study (30 ‘spared’ plans, 30 ‘non-spared’ plans; Fig. 3). For cases with unacceptable major dose deviations, additional non-coplanar treatment plans were created.

For the analysis, dose/volume metrics following ICRU 91 [30] of target volumes and organs at risk as well as plan metrics (starting gantry beam angle, monitor units [MU], conformity indices of the 95 % [CI95] and 50 % [CI50] isodose) were exported from Monaco to Microsoft Excel (Microsoft Corporation, US). Instead of *D*_max_, *D*_0.035cm_^3^ was documented as point dose (ICRU 91). The smallest distance between the S-ICD device and the nearest point of each target volume (S8, S11, S17) was determined in Monaco.

### 2.4 Statistical analysis

Statistical analysis was performed with SPSS (Chicago, IL, USA; version 28). For each segment, basic statistical parameters (median, interquartile range [IQR]) of spared and non-spared plans were gathered. Differences of dose/volume and plan metrics between spared and non-spared plans were evaluated with Wilcoxon Signed Rank Test (p < 0.05). For comparison of differences of dose/volume and plan metrics between the location of the target volumes (S8, S11, and S17), Friedman’s test was used.

## 3 Results

### 3.1 Dose to S-ICD

S-ICD constraints could be fulfilled in 27 of 30 spared plans (Table 1). Median *D*_0.035cm_^3^ to the S-ICD coil was significantly higher for spared plans with the PTV in S11 compared to non-spared plans (4.8 Gy [IQR 4.0 Gy] vs. 2.1 Gy [IQR 0.8 Gy], p = 0.02), respectively. For median *D*_0.035cm_^3^ in plans with the PTV in S8 (7.1 Gy [IQR 1.1 Gy] vs. 6.7 Gy [IQR 2.1 Gy]) or S17 (4.7 Gy [IQR 3.9 Gy] vs. 4.4 Gy [IQR 1.2 Gy]), no significant differences between spared and non-spared plans occurred. In Friedman’s test, there was a significant difference for both spared (p = 0.03) and non-spared (p < 0.01) plans between the three PTV locations. The median shortest distance between the S-ICD and the PTV was 9.3 cm (range 8.0–12.2 cm) for S8, 4.6 cm (range 1.0–6.8 cm) for S11, and 6.8 cm (range 3.8–11.3 cm) for S17.

**Table 1:** Comparison of dose/volume metrics for plans for all three representative segments with sparing of the S-ICD from the primary beam (‘spared’) vs. plans without considering the S-ICD during treatment planning (‘non-spared’). All values as median (IQR) for the results of ten patients (significance level p < 0.05).

	**Segment 8**			**Segment 11**			**Segment 17**		
**Dose/volume metric**	**Spared plan (n = 10)**	**Non-spared plan (n = 10)**	***P-value***	**Spared plan (n = 10)**	**Non-spared plan (n = 10)**	***P-value***	**Spared plan (n = 10)**	**Non-spared plan (n = 10)**	***P-value***
**S-ICD** *D_0.035ccm_^3^* (Gy)	0.4 (0.1)	5.1 (4.3)*	***0.01***	0.4 (1.1)	8.5 (2.6)*	***0.01***	0.4 (0.1)	2.9 (4.2)*	***0.02***
**CTV** *D_0.035cm_^3^* (Gy)	30.0 (0.6)	31.0 (2.0)*	***0.02***	30.1 (0.9)	30.8 (0.9) *	*0.11*	29.6 (0.9)	29.6 (0.8)*	*0.72*
**PTV** *D_2%_* (Gy)	29.6 (0.8)	30.5 (2.1) *	***0.01***	29.4 (0.8)	30.2 (0.7) *	*0.07*	29.3 (0.8)	29.3 (0.7) *	*0.72*
**PTV** *V_25Gy_* (%)	95.0 (0.1)	95 (0.0)	*0.83*	95.0 (0.1)	95.0 (0.8)	*0.07*	95.0 (0.1)	95.0 (0.2)	*0.46*
**Aorta** *D_0.035cm_^3^* (Gy)	13.3 (5.9) *	9.6 (7.5) *	***0.04***	9.9 (3)*	7.3 (1.8) *	***0.01***	2.6 (0.9) *	1.9 (0.6) *	***0.02***
**Thoracic wall***D_0.035cm_^3^* (Gy)	17.8 (5.8) *	16.7 (5.6) *	*0.06*	16.7 (7.6) *	12.8 (5.3) *	***0.01***	26.5 (9.7) *	26.8 (11.6) *	*0.88*
**Heart-PTV***D_50%_* (Gy)	4.1 (1.2) *	3.6 (1.9) *	***0.01***	0.8 (0.8) *	1.5 (0.9) *	***0.01***	0.4 (0.9) *	0.3 (0.6) *	***0.01***
**LAD** *D_0.035cm_^3^* (Gy)	26.8** (4.9)*	26.5** (11.2)*	*0.72*	9.4 (4.9) *	4.9 (2.5) *	***0.01***	20.9** (14.6)*	21.5** (17.2)*	*0.65*
**Left lung** *V_10Gy_* (%)	0.2 (1.0) *	0.1 (0.7) *	*0.24*	4.5 (3.8) *	4.3 (3.0) *	***0.03***	0.0 (0.4) *	0.1 (0.5) *	*0.35*
**Left lung** *V_5Gy_* (%)	1.9 (3.2) *	2.9 (3.3) *	***0.01***	13.8 (6.3) *	13.3 (7.7) *	*0.17*	0.9 (1.9) *	0.8 (1.3) *	*0.48*
**Right lung** *V_10Gy_* (%)	0 (0)	0 (0)	*1.00*	0 (0)	0 (0)	*0.32*	0 (0)	0 (0)	*1.00*
**Right lung** *V_5Gy_* (%)	1.1 (1.9) *	0.2 (0.5) *	***0.01***	1.6 (3.0) *	0 (0) *	***0.01***	0 (0.1) *	0 (0) *	*0.18*
**Spinal canal** *V_6Gy_* (cc)	0 (0)	0 (0)	*1.00*	0 (0)	0 (0)	*0.32*	0 (0)	0 (0)	*1.00*
**Spinal canal***D_0.035cm_^3^* (Gy)	1.4 (0.5) *	1.5 (0.4) *	*0.96*	4.2 (1.4)*	2.9 (1.0) *	***0.02***	1.2 (0.6) *	0.8 (0.5) *	***0.01***
**Skin** *V_10Gy_* (cm^3^)	0 (0.0) *	0 (0)	*0.41*	0.11 (2.6) *	0 (0)	***0.01***	0.6 (1.3) *	0 (0.2)	***0.02***
**Skin** *D_0.035cm_^3^* (Gy)	8.3 (1.6) *	8.0 (1.2)	*0.09*	10.9 (4.2)*	7.5 (1.6)	***0.05***	11.1 (3.7)*	7.9 (3.6)	***0.01***
**Esophagus** *V_9Gy_* (cm^3^)	0 (0) *	0 (0)	*1.00*	0.3 (0.8)*	0 (0.0)	***0.02***	0 (0) *	0 (0)	*1.00*
**Esophagus***D_0.035cm_^3^* (Gy)	3.5 (0.8) *	2.8 (1.2) *	*0.09*	9.3 (3.3) *	5.9 (2.3) *	***0.01***	2.4 (0.7) *	2.0 (1.3) *	*0.29*

* Significant difference (Friedman’s test, p < 0.05) of this median dose/volume metric compared to the other PTV segments within the protocol group (spared or non-spared).

** Major dose deviation to LAD D_0.035cm_^3^ due to location in the PTV, therefore dose deviation clinically acceptable.

Minor protocol deviations/dose constraints (RAVENTA): ICD 0.5 Gy < D_max_ ≤ 1.0 Gy; CTV D_max_ ≥ 30 Gy; PTV D_2%_ ≤ 32.5 Gy; PTV V_25Gy_ ≥ 95 %; Aorta 20 Gy < D_max_ ≤ 25 Gy; Whole heart – PTV D_50%_ ≤ 5 Gy; LAD 14 Gy < D_max_ ≤ 20 Gy; Spinal canal D_max_ ≤ 8 Gy, V_6Gy_ ≤ 1 cm^3^; Skin D_max_ ≤ 16 Gy; Esophagus D_max_ ≤ 19 Gy, V_9Gy_ ≤ 4 cm^3^ (Blanck et al., Clin Res Cardiol, 2020). For all other structures, general dose constraints for stereotactic radiotherapy were considered: Thoracic wall D_0.035cm_^3^ ≤ 30 Gy (Gerhard et al., Pract Radiat Oncol, 2021); Lung V_10Gy_ ≤ 10 %; Lung V_5Gy_ ≤ 35 % (Sahgal et al., Lancet Oncol, 2021).

Abbreviations: S-ICD = Subcutaneous Implantable Cardioverter Defibrillator, CTV = Clinical Target Volume, LAD = Left Anterior Descending Artery, PTV = Planning Target Volume.

### 3.2 Comparison of dose/volume metrics

Median PTV volume was 37.6 cm^3^, 38.5 cm^3^, and 14.8 cm^3^ for S8, S11, and S17, respectively. In general, the PTV dose coverage was clinically acceptable with PTV *V_25Gy_* ≥ 95 % in 32 plans and minor deviations in 28 plans with PTV *V_25Gy_* between 93, 9% and 95 %. However, when analyzing each segment (S8, S11, and S17), some dose/volume metrics of organs at risk showed significant differences between spared and non-spared plans (Table 1). Major dose deviation to the LAD in plans with PTV in S8 and S17 was unavoidable due to the anatomic proximity of the LAD and the PTV (LAD location inside the PTV and CTV of S8 in eight and three, S11 in zero and zero, and S17 in five and one cases, respectively). In the RAVENTA study protocol, such unavoidable major dose deviations to the LAD are clinically acceptable. Dose to other organs at risk than LAD or S-ICD was clinically acceptable in most cases, with no minor dose deviation in S8 plans and only one minor dose deviation (skin *D_0.035cm_^3^* 15.6 Gy) in S17 plans. Major dose deviations to other organs at risk than LAD or S-ICD only occurred in one out of 60 plans (PTV S11, see 3.4).

### 3.3 Comparison of plan metrics

In the analysis of plan metrics, significant differences between spared and non-spared plans could be observed with better conformity and lower count of MU for non-spared plans **(**Table 2**)**. Additionally, all median plan metrics showed a significant difference between the three PTV locations in Friedman’s test for spared as well as for non-spared plans (p < 0.05).

**Table 2:** Comparison of plan metrics for plans for all three representative segments with sparing of the S-ICD from the primary beam (‘spared’) vs. plans without considering the S-ICD during treatment planning (‘non-spared’). All values as median (IQR) for the results of ten patients (significance level p < 0.05).

	**Segment 8**			**Segment 11**			**Segment 17**		
**Plan metric**	**Spared plan (n = 10)**	**Non-spared plan (n = 10)**	***P-value***	**Spared plan (n = 10)**	**Non-spared plan (n = 10)**	***P-value***	**Spared plan (n = 10)**	**Non-spared plan (n = 10)**	***P-value***
Starting Gantry Beam Angle	−120° (13°)	−70° (20°)	***0.00***	−130° (20°)	20° (23°)	***0.01***	−110° (13°)	−65° (13°)	***0.01***
Monitor Units	6325 (787)	5933 (959)	*0.58*	6533 (2706)	6114 (1170)	*0.33*	4917 (1447)	4536 (920)	***0.01***
CI95	1.2 (0.0)	1.1 (0.1)	***0.04***	1.2 (0.1)	1.1 (0.1)	***0.01***	1.3 (0.1)	1.2 (0.1)	***0.02***
CI50	3.7 (0.4)	3.4 (0.3)	*0.06*	5.1 (1.8)	3.6 (0.4)	***0.01***	5.6 (1.6)	3.8 (0.4)	***0.01***

Abbreviations: CI95 = Conformity Index of the 95 % isodose, CI50 = Conformity Index of the 50 % isodose.

### 3.4 Individual cases with major dose deviation to S-ICD

**Fig. 4:**
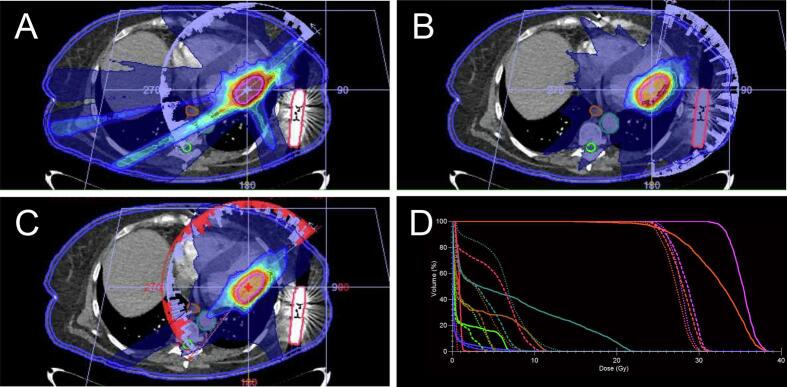
Exemplary treatment plans with clinically unacceptable spared plan (sparing of the S-ICD). A) Radiotherapy plan with sparing of the S-ICD from the primary beam for the PTV in segment 11 (‘spared’ plan). Note the direct proximity of the S-ICD to lateral parts of the left ventricle. B) Radiotherapy plan for the same patient and segment without sparing of the S-ICD from the primary beam (‘non-spared’). In this case, sparing of the S-ICD resulted in plan compromises with major/unacceptable protocol deviations by means of S-ICD D_0.035cm_^3^ over 1 Gy, aorta D_0.035cm_^3^ over 20 Gy, spinal canal D_0.035cm_^3^/V_6Gy_ over 8 Gy/ > 1 cm^3^, skin D_0.035cm_^3^ over 16 Gy, and esophagus V_9Gy_ over 4 cm^3^. C) Spared non-coplanar radiotherapy plan. With non-coplanar treatment planning all major protocol deviations could be avoided. Only PTV V_25Gy_ resulted in a minor protocol deviation of 90,1 %. D) Dose/volume histogram of all structures with major protocol deviations in the spared coplanar radiotherapy plan in comparison with the non-spared coplanar and spared non-coplanar radiotherapy plan. Legend: Solid line = spared coplanar radiotherapy plan, dashed line = non-spared coplanar radiotherapy plan, dotted line = spared non-coplanar radiotherapy plan. Orange = PTV S11, purple = CTV S11, red = S-ICD, turquoise = aorta, green = spinal canal, brown = esophagus, blue = skin. (For interpretation of the references to colour in this figure legend, the reader is referred to the web version of this article.)

A major protocol violation by means of dose deviation to the S-ICD in spared plans with the PTV in S11 was unavoidable in three out of ten cases (*D*_0.035cm_^3^ 1.9 Gy, 1.8 Gy, and 1.3 Gy). Distance between the PTV and the S-ICD in these cases was 3.7 cm, 1.0 cm, and 3.0 cm, due to special anatomical situation caused by cardiomegaly and mediastinal shift, respectively. Additional clinically not acceptable major protocol deviations for case one (Fig. 4) were PTV *D*_2%_ (37.6 Gy), spinal canal *V*_6Gy_ (3.8 cm^3^), as well as esophagus *V*_9Gy_ (4.9 cm^3^). Additionally, minor deviation occurred in following metrics: aorta *D*_0.035cm_^3^ (22.2 Gy), spinal canal *D*_0.035cm_^3^ (7.5 Gy), and skin *D*_0.035 cm_^3^ (15.9 Gy). For case two, only PTV *V*_25Gy_ resulted in a minor protocol deviation (94.0 %). In case three, all other dose constraints were acceptable. In comparison to median MU for spared plans of S11, MU in these three cases were higher (case one: 14,486 MU, case two: 7677 MU, case three: 8219 MU). CI95 was 1.3 for all cases, CI50 was 8.2 for case one, 5.9 for case two, and 6.4 for case three, respectively.

Non-coplanar treatment planning improved the plan quality with less dose to structures at risk: For case one, only a minor protocol deviation to the S-ICD (*D*_0.035cm_^3^ 0.7 Gy) and PTV *V*_25Gy_ (90.1 %) remained. A major deviation to the S-ICD was persistent in case two with *D*_0.035cm_^3^ of 1.4 Gy, accompanied by a minor deviation of PTV *V*_25Gy_ with 90.1 %. S-ICD *D*_0.035cm_^3^ for case three was 0.8 Gy with non-coplanar treatment planning (minor deviation) without further protocol violations. MU were lower for case one (11699 MU) and two (5993 MU), and higher for case three (14067 MU) compared to coplanar plans. CI95 was 1.1 for case one, 1.3 for case two, and 1.2 for case three, with a CI50 of 3.7, 5.3, and 5.2, respectively.

## 4 Discussion

In this retrospective treatment planning study, the impact of an implanted S-ICD on radiation dose distribution was evaluated by creating and comparing two types of treatment plans (with and without sparing of the S-ICD) for three hypothetical PTVs. The S-ICD was successfully spared in 27 out of 30 plans. However, in some cases, sparing led to reduced plan quality, particularly with a PTV in lateral heart segments.

Suspected malfunction of ICDs due to high single-dose radiation includes transient device upsets during treatment (i.e., inappropriate shock), software malfunction, particularly affecting the random access memory, or permanent damage (i.e., battery depletion) [31]. For conventional hyper-fractionated radiotherapy, recommendations on maximal ICD doses have been published based on ex-vivo data of irradiated explanted devices [16]. Exact dose constraints to the ICD device and leads, especially for a high single-dose STAR, are not known; however, a consensus exists that the dose should be minimized, and most study protocols recommend reducing the dose below 1 Gy. Cardiac implantable electronic devices are particularly sensitive to neutron-induced effects at higher photon energies, and must be carefully evaluated prior to radiotherapy to prevent electronic malfunction. This is emphasized in current expert consensus and guidelines [[32], [33], [34]]. In a retrospective study of patients receiving thoracic stereotactic ablative radiotherapy Levis et al. saw no inappropriate events in 34 patients (24 with a pacemaker and ten with an ICD) with a dose to the ICD < 2 Gy and the beam energy ≤ 10 MV [35]. In an analysis of 43 patients undergoing STAR, no major ICD malfunction or clinically relevant alterations in ICD dose/volume metrics were observed, with a median ICD generator dose of 0.1 Gy and median lead tip doses of 6.8 Gy (right ventricle), 6.7 Gy (left ventricle), and 2 Gy (right atrium) [15]. Additionally, the authors report that one ICD reset was observed in the limited follow-up time of twelve months. The first reported case of a cardiac implantable electronic device complication was published in 2023, with a significant rise in the right ventricular lead threshold from 1.5 V to 2.63 V (1 ms pulse width) at a maximum dose to the RV lead tip of 6.8 Gy [31]. In this study, a feasible and safe plan that is in line with best practice recommendations on STAR treatment planning [29] and fulfills the RAVENTA study recommendations with maximal minor protocol deviations could be generated in 27/30 plans, despite the anatomical proximity of the target volume to the S-ICD. Median dose to the S-ICD coil in spared plans ranged from 4.7 Gy (PTV in S17) to 7.1 Gy (PTV in S8) which is comparable with the value reported above that lead to a significant rise in the ventricular lead threshold.

For rare cases with laterally localized targets and anatomical variations (e.g., cardiomegaly) resulting in a distance between PTV and S-ICD < 4 cm and consecutive major dose deviation to the S-ICD, an individualized, interdisciplinary strategy is needed. Advanced treatment planning techniques such as non-coplanar planning, which has been thoroughly evaluated for lung stereotactic body radiotherapy, can improve plan quality in these cases [36,37]. If a major dose deviation to the S-ICD is still unavoidable, one possible solution would be to accept this protocol violation and perform close cardiac follow-ups after STAR. This way, malfunctions of the S-ICD could be recognized, and early intervention or device revision could be evaluated. If this strategy is clinically unacceptable, another solution consists of re-implantation of a different, transvenous system implanted pectorally prior to STAR. However, in this case, the patient must undergo another intervention with perioperative risks such as bleeding, infection, or complications from anesthesia [38]. S-ICD relocation, therefore, should only be performed if all plan optimization methods for STAR, as well as all treatment alternatives, have been exhausted.

Differences between plan metrics were in concordance with observed differences in dose/volume metrics. Although there were no significant differences between MUs for spared and non-spared plans, a significant difference could be observed for CI95 as well as CI50. A higher CI95 in spared plans indicates less conformity of the 95 % isodose line with the PTV, while a higher CI50 indicates less conformity of the 50 % isodose line with the PTV, and therefore flatter dose gradients with higher low dose radiation to the patient outside the PTV [39]. During planning, spared plans were more complex due to sparing of the S-ICD. The analyzed plan metrics reflect this impression during treatment planning, with reduced plan quality for spared plans compared to non-spared plans. Especially in the three individual cases with a major dose deviation to the S-ICD, a simultaneous reduction of plan metrics with higher MUs, CI95, and CI50 could be observed.

One limitation of this study is the small number of patients included in this analysis (n = ten). Moreover, these patients were not STAR patients but rather a selection of S-ICD patients treated at our university hospital. For the selected patients, no further data on clinical details or follow-up are available. Additionally, the hypothetical PTV created for this planning study were based on only one segment of the American Heart Association 17-segment model. The resulting PTVs were therefore rather small compared to PTVs used in patients for treatment with STAR (median PTV volume ranging from 14.8 to 38.5 cm^3^ compared to median irradiated volumes of 52.2 to 198.3 cm^3^ at study level and 80.6 cm^3^ in meta-analyses) [12,40]. Consequently, the dose to organs at risk could increase when larger PTVs are assumed [41].

Based on this analysis of the feasibility of STAR treatment in patients with an S-ICD, one future research project would be to use existing treatment plans from patients with an S-ICD treated with STAR within the STOPSTORM consortium. With such a larger dataset of STAR patients with an S-ICD or also ICD in general, the further dosimetric impact of (S-)ICD sparing, clinical data regarding post-STAR (S-)ICD function, as well as a proposition of dose constraints for the (S-)ICD device/coils/leads could be evaluated.

In this retrospective planning study, the S-ICD could be spared in 27 out of 30 plans, despite individual differences in device and lead position. The presence of an S-ICD is not a contraindication for STAR. Rare anatomical situations with a distance between the PTV and the S-ICD of <4 cm can pose challenges in radiation treatment planning. A future analysis of S-ICD function in a large dataset of STAR patients is needed to conduct dose recommendations for the S-ICD device and coil.

## CRediT authorship contribution statement

**Lena Kaestner:** Data curation, Investigation, Methodology, Visualization, Writing – original draft. **Jingyang Xie:** Investigation, Methodology, Software, Visualization, Writing – review & editing. **Hannah Fanslau:** Investigation, Writing – review & editing. **Kerstin Siebenlist:** Investigation, Writing – review & editing. **Miriam Eckl:** Data curation, Investigation, Writing – review & editing. **Hans Oppitz:** Data curation, Software. **Jens Fleckenstein:** Conceptualization, Data curation, Investigation, Software, Supervision. **Daniel Buergy:** Supervision, Writing – review & editing. **Mustafa Kuru:** Writing – review & editing, Investigation. **Jürgen Kuschyk:** Investigation, Software. **Daniel Dürschmied:** Resources, Supervision, Writing – review & editing. **Mathieu Kruska:** Investigation, Writing – review & editing. **David Krug:** Writing – review & editing. **Frank A. Giordano:** Resources, Supervision, Writing – review & editing. **Achim Schweikard:** Resources, Software, Supervision, Writing – review & editing. **Oliver Blanck:** Supervision, Writing – review & editing. **Boris Rudic:** Conceptualization, Formal analysis, Methodology, Project administration, Writing – original draft. **Judit Boda-Heggemann:** Conceptualization, Data curation, Methodology, Project administration, Writing – original draft.

## Declaration of competing interest

The authors declare the following financial interests/personal relationships which may be considered as potential competing interests: **LK:** Personal fees from AstraZeneca, outside of the submitted work. **DB:** Personal fees from NB Capital ApS, personal fees from b.e. Imaging GmbH, personal fees from PharmaMar, outside of the submitted work. **FAG:** Grants and personal fees from Carl Zeiss Meditec AG, grants and personal fees from TME Pharma AG, personal fees from Guerbet SA, personal fees from Cureteq AG, personal fees from Bristol-Myers Squibb, personal fees from AstraZeneca GmbH, personal fees from FoMF GmbH, from MEDAC GmbH, from Elsevier GmbH, other from TME Pharma AG, other from Implacit GmbH, outside of the submitted work. **OB:** Section editor for medical physics of the Strahlentherapie und Onkologie journal, outside of the submitted work. **JBH:** Personal fees from EBAMed SA, grants from Elekta AB, personal fees from AstraZeneca, outside of the submitted work. All other authors declare that they have no competing interests.
